# Design and implementation characteristics of research training for rural health professionals: a qualitative descriptive study

**DOI:** 10.1186/s12909-023-04169-5

**Published:** 2023-03-30

**Authors:** Claire Quilliam, Anna Wong Shee, Denise Corboy, Kristen Glenister, Olivia King, Kevin Mc Namara, Laura Alston, Drew Aras, Alison Beauchamp, Carol McKinstry

**Affiliations:** 1 Department of Rural Health, The University of Melbourne, 49 Graham Street, Shepparton, VIC 3630 Australia; 2 Grampians Health, 102 Ascot St Sth, Ballarat, VIC 3350 Australia; 3 Deakin Rural Health, Deakin University, Princes Hwy, Warrnambool, VIC 3280 Australia; 4 Blue Sky Mind Research Consultancy, Lake Wendouree, Victoria, 3350 Australia; 5 The University of Melbourne, Docker Street, Wangaratta, VIC 3677 Australia; 6 Western Alliance Academic Health Science Centre, 25 Ryot Street, Warrnambool, VIC 3280 Australia; 7 The Global Obesity Centre, Institute for Health Transformation, Deakin University, 1 Gheringhap Street, Geelong, VIC 3220 Australia; 8 Research Unit, Colac Area Health, 2-28 Connor St, Colac, VIC 3250 Australia; 9 Monash Rural Health, Monash University, 15 Sargeant St, Warragul, VIC 3820 Australia; 10 La Trobe University, Edwards Rd, Flora Hill, Victoria, 3552 Australia

**Keywords:** Rural population, Health occupations, Rural health services, Research, Capacity building, Education

## Abstract

**Background:**

Research capacity and capability of rural health professionals is essential to the delivery of evidence-based care and for informing strategies to address rural health inequities. Effective implementation of research education and training is fundamental to building rural health professional research capacity and capability. A lack of overarching guidance to inform the delivery of research education and training in rural health services can contribute to gaps in capacity-building approaches. The aim of this study was to identify characteristics of the design and implementation of current research training for rural health professionals in Victoria, Australia, to inform a future model for rural health professional research capacity and capability building.

**Methods:**

A qualitative descriptive study was undertaken. Key informants, with extensive knowledge of research education and training in rural health services in Victoria, were invited to participate in semi-structured telephone interviews via snowballing recruitment methods. Interview transcripts were analysed inductively, with themes and codes mapped to the domains of the Consolidated Framework for Implementation Research.

**Results:**

Of the 40 key informants approached, 20 agreed to participate including 11 regional health service managers, five rural health academics and four university managers. Participants suggested that research training varied in quality and relevance to rural health professionals. Training costs and lack of tailoring to the rural context were key barriers, whereas experiential learning and flexible modes of delivery enabled training uptake. Health service and government policies, structures, and processes both enabled or stifled implementation opportunities, with rural health professional networks from different regions offering capacity for research training development, and government departmental structures hampering training coordination. Tension between research activities and clinical practice, and health professional knowledge and beliefs, shaped the delivery of training programs. Strategically planned and evaluated research training programs and education via co-design with rural health professionals and use of research champions were strongly recommended by participants.

**Conclusions:**

To optimise research training for rural health professionals and increase the quality and quantity of relevant rural health research, a systematically planned, implemented, and resourced region-wide research training model is required.

## Background

Building the research capacity and capability of rural, regional, and remote health professionals and health services is key to promoting evidence-based practice and reducing health inequalities between non-metropolitan and metropolitan communities [1–5]. Research improves health outcomes although historically, research has been greatly under-invested within the rural context [6]. For the purposes of this study, the term ‘rural’ will be used to refer to both rural and regional areas. In the Australian context, publicly funded health services and related budgets, including those for research, are primarily managed by state and territory departments [7]. However, commonwealth funding has supported implementation of rural and regional research education and training, among other capacity building mechanisms, primarily through Rural Health Multidisciplinary Training (RHMT) program funding. The recent evaluation of the RHMT highlighted the need for high-quality research training and research support for local health professionals and broader community stakeholders [8].

Research education and training is the cornerstone of research capacity and capability building strategies for health professionals [9]. *Research education and training* is a broad concept that typically encompasses discrete initiatives or interventions where curriculum is delivered to individuals or groups to build research skills [10, 11] and includes other mechanisms where health professionals receive research education and training, for example, via embedded researchers [12] or being involved in the research process [13]. The approaches to developing and delivering research education and training to health professionals differ across organisations and jurisdictions [10]. The nature of previous research capacity and capability building strategies implemented in rural areas of Australia have been influenced by context-specific factors and challenges such as health workforce shortages [2, 14, 15], and inadequate funding for research capacity and capability building and research activity [2, 3, 15, 16]. As a result, there are state-based differences in approaching and coordinating the delivery of research education and training to rural health professionals [5, 17]. The peer-reviewed literature on approaches to research education and training varies between Australian States and Territories, with little literature specific to Tasmania, Northern Territory, Canberra, and South Australia. The state funded approaches taken in four Australian States, including Western Australia, Queensland, New South Wales and Victoria, are briefly summarised below.

The Western Australian Health Translation Network (WAHTN) is a state-wide National Health and Medical Research Council (NHMRC) accredited collaborative comprising health services, universities, medical research institutes, and other private and public health organisations. Although the Western Australian Government does not offer a state-wide research training program specifically for rural health professionals, the WAHTN hosts the online Research Education and Training Program available to staff working in public and private health organisations across the State [18].

Queensland state-funded researchers have been embedded in hospital and health services since 2010, dispersed across 11 organisations, primarily in metropolitan and regional locations [19]. Individual hospitals and health services develop and deliver bespoke research training programs via the state-funded embedded health professional researchers [16, 19]. While there is little coordination of foundational research training between services, there is a state-supported online resource and tele-mentoring program (known as Allied Health – Translating Research into Practice [AH-TRIP]) to promote the translation of research into practice [20, 21]. The AH-TRIP mentoring component is offered via an expression of interest to all health professionals in Queensland Health funded hospital and health services, including rural health services. In 2019, the Health Practitioners and Dental Officers Certified Agreement included dedicated and recurrent funding for building research capacity allocated to Queensland rural hospital and health services [22].

In New South Wales, the Rural Research Capacity Building Program (RRCBP) was established in 2006. The RRCBP is a state-funded two-year mentored training program that aims to increase rural health research capacity [4, 17, 23]. The program is open to experienced health workers in rural, publicly funded health services. Entry is via a competitive process whereby applicants are required to submit an organisationally endorsed, viable research proposal [4]. The RRCBP and a related rural research capacity-building initiative have been evaluated formally, demonstrating positive participant-reported outcomes, organisational benefits, and increased research outputs [4, 17, 24, 25]. As a competitive process, not all applicants are accepted into the annual program, however it represents a coordinated approach to rural health research training across the rural health districts.

In Victoria, publication of the Allied Health Victorian Research Framework in 2018 provided a platform for the development and evaluation of research capacity and capability building strategies. The Framework outlines the implementation of the Allied Health Clinical Educators Network (AHCEN) and the Allied Health Research and Translation (AHRT) program [26]. The Framework describes the role of the AHCEN in supporting the translation of research into practice, and the role of AHRT facilitators in building research capacity within and across health organisations. The delivery of research training is often considered an organisation-led action, lacking an emphasis on systematic, coordinated delivery of research training programs and rural specific/tailored initiatives. The Framework and the implementation of clinical educator and AHRT facilitation roles are focused on allied health professional education and there are no equivalent frameworks or programs for nursing or medical professionals.

There is a need for a strategic, coordinated approach to research education for all health professionals in Victoria, particularly in rural health services. The paucity of knowledge surrounding the characteristics of research education and training delivered across Victoria and other Australian jurisdictions, particularly relating to how training is developed and implemented, warrants further research attention. A more detailed understanding of current training characteristics will enable further development of an evidenced-based research training model that can be delivered with consistency to support rural health services and health professionals to conduct locally-driven research, in Australian states and internationally. The aim of the study was to identify the design and implementation characteristics of current research training for rural health professionals in Victoria to inform a future research training model that will build rural health professional research capacity and capability.

## Methods

### Design

A qualitative descriptive research methodology was used as the research design for this study [27]. A Human Research Ethics Committee from Ballarat Health Services and St John of God Healthcare granted ethics approval for this study [LNR/60139/BHSSJOG-2019-196011(v1)]. In this study, the term ‘rural’ includes areas that are classified as Modified Monash Model categories 2–7 (large regional centres to very remote communities) [28].

### Participants

Participants with a knowledge of research education and training and the current status of health research in rural Victoria were recruited. Participants were required to have lived in rural Victoria or have recent experience working in rural Victoria for at least three consecutive years. Relevant work experience included health professional education, health-related research, or public health. A snowball sampling approach was used to recruit potential participants, via researcher networks and personal communication, internet searches of peak body websites and journal websites, and grey literature searches. Forty potential participants were identified and provided with a plain language statement via email, then invited to participate in a telephone interview. Twenty participants agreed to be involved and provided written and verbal consent prior to interview. The participant sample included 11 rural health managers, five rural health academics/research coordinators, and four university sector directors/managers. Participants came from organisations with catchment populations ranging between 5,000 to over 250,000, and from various regions of Victoria ranging from Modified Monash categories (MM) 2–5 (large regional centres and large, medium and small rural towns).

### Data collection

Researcher DC, who conducted the semi-structured telephone interviews, had no prior working or personal relationships with participants. The interviews occurred between February and March 2020 and ranged between 30 and 86 min, averaging 49 min. Cooke’s [29] Framework for Evaluating Research Capacity Building in Healthcare informed development of the interview guide questions and prompts, particularly around research supports and partnerships, research training infrastructure, and characteristics that enabled effective and sustainable training models. Cooke’s framework has informed and successfully supported the robustness of research activity within other research education studies [30, 31]. The interview guide comprised eight questions relating to research training and research capacity building, for example: “Can you tell me about the opportunities for health professionals in your area to engage in health research education?” Handwritten notes were taken during the interviews and audio recordings were transcribed verbatim.

### Data analysis

Interview transcripts were analysed inductively, using Braun and Clarke’s [32] thematic analysis techniques. This process involved researcher DC becoming familiar with the data, generating codes from the data, and organising codes to form potential themes. Researchers DC, CQ and AWS discussed and clarified themes in relation to the data and codes, and finalised themes. The inductive analysis process generated 46 codes, 10 minor themes and two overarching themes: *doing research*, and *research training*. The developed codes and associated data within the research training overarching theme were then mapped to the Consolidated Framework for Implementation Research (CFIR) [33] to identify design and implementation characteristics of research training for rural health professionals.

The CFIR is commonly used in health research to explore the influence of intervention characteristics, and also organisational and environmental context, on implementation [33–35]. The CFIR comprises 39 constructs within five overarching domains: intervention characteristics, outer setting, inner setting, characteristics of individuals, and process [33], although not all constructs need to be identified when the CFIR is used in implementation studies [36]. After researchers CQ, AWS, OK and DC independently mapped the codes and associated data against the CFIR domains and constructs, they met to discuss the mapping process, identify relevant constructs and resolve disagreements.

## Results

Researchers identified 15 relevant constructs within the five CFIR domains related to the design and implementation characteristics of research training for rural health professionals (see Table 1 for exemplar constructs, codes and quotes): intervention characteristics (intervention source, cost, adaptability and complexity); outer setting (cosmopolitanism, external policies and incentives); inner setting (relative priority, leadership engagement, organisational incentives and rewards, access to knowledge and information); characteristics of individuals (knowledge and beliefs about the intervention, individual stage of change); and process (planning, engaging, reflecting and evaluating). Findings and supporting quotes are provided under the five CFIR domain headings and italicised constructs, along with the Modified Monash category (MM) associated with the contributing participant.

**Table 1:** CFIR domains, exemplar constructs, codes and quotes

*Domain*	*Exemplar construct*	*Exemplar code and quote*
Intervention characteristics	Intervention source	Quality of research training: “I think it’s [research training] made way too heavy continuously. It’s classroom taught rather than through experientially.” (Participant 6, MM4)
Outer setting	Cosmopolitanism	Sharing of resources among regional organisations: “Another challenge, if we’re looking at more targeted programs that people are interested in undertaking research, is the numbers of people that will be interested in rural and regional areas. I think there would be great benefits from different regions collaborating to try and have a critical mass of numbers to make some of these research programs sustainable.” (Participant 3, MM2)
Inner setting	Relative priority	Other educational commitments as a barrier: “You really have to be focused on the processes of the health service, meeting all of your appointment needs, keeping up with your own professional development at the same time. There’s no wiggle room at all to take on anything extra.” (Participant 4, MM3)
Characteristics of individuals	Knowledge and beliefs about the intervention	Staff interest in training: “I think there is a fair bit of interest among clinicians to do research, to do something, otherwise their work can get quite boring because they’re doing the same thing over and over again.” (Participant 7, MM4)
Process	Engaging	Need opportunity to apply skills learned: “It [training] has to be part of a broader program of linking them [health professionals] in with academics and other organisations… They're going to learn about what a research project looks like and how they can be involved, but then where to from there? What are they going to do with that information?” (Participant 16, MM4)

### Intervention characteristics

Participants described various research training opportunities for health professionals in rural Victoria. These included half-day sessions, online short courses, week-long intensives, longer-term support programs and university higher degree programs. Some participants noted the impromptu nature of training, driven by the “need of the day” (Participant 9, MM2), particularly with the training topic: “It’s been a bit ad hoc in the past… [We’ve had] sessions around finding evidence or appraising evidence, et cetera but nothing consistent” (Participant 3, MM2).

Where the research training was developed or *sourced* was considered critical to the successful uptake and implementation of research training programs. Participants noted that most of the training available was developed in metropolitan areas: “all of the big hospitals are in Melbourne [capital city, state of Victoria], and all of the complex cases are transferred to Melbourne, most of the [research] training is held by the Melbourne hospitals” (Participant 1, MM3). Metropolitan-based training programs, where complex research methods such as statistical analysis are sometimes favoured, were not always considered relevant by rural health professionals when conducting small scale exploratory research: “for rural [health professionals], we engage more in social research, and maybe we need to look at it from a different perspective” (Participant 19, MM5). Training delivered by university academics or that mirrored university undergraduate courses often poorly reflected the day-to-day reality of conducting research in rural health contexts:

It's not earthshattering. You bring in a university academic. They want to talk about research paradigms, and different methods. They [clinicians] want to talk about rehab generally. Is this the right rehab or whatever? I think the clinician gets overwhelmed with that. They [clinicians] just want to know [whether to provide rehabilitation for] four months or six months. (Participant 2, MM3)

Many training programs developed externally to rural communities and health services were considered inaccessible and resulted in rural health professionals feeling unsure about their capacity to complete the training or conduct research:

There are so many things [training opportunities] that are available, but people won’t take them up because they don’t see the relevance and they don’t feel safe… You’ve just got to take their hand to the edge of the water and say … ‘all you’re doing today is dipping your toe in the water. That’s all.’ (Participant 19, MM5)

The *cost* and lack of available funding impacted opportunities for research training to be implemented for rural health professionals. Competitive funding appeared to favour metropolitan-based health services. Rural health services or individual rural health professionals were often expected to fund training attendance fees in addition to backfill for staff absence, travel and accommodation costs associated with attending training:

They [a statewide health service] have all these free workshops but then it's a matter of someone having to get the day off and spend the night before in Melbourne. For us, that's no longer a free option; it's still a cost. (Participant 17, MM3)

Rural health services that delivered training struggled to recover costs given the expectation they offer reduced or no attendance fees:

You might have the Department of Epidemiology [in Melbourne] carry out a one or two-day course for health professionals; $500, $1000, $1500 or whatever it is, and people do it. …Whereas [in] the rural [communities], I find that you can’t charge that kind of money because basically no one would actually do the course. In a lot of cases, we don’t charge anything at all, or minimal costs just to cover food. Even then, we virtually moved to not charging anything because we found no one was willing to pay. (Participant 12, MM4)

Participants highlighted that research training could be better *adapted* to the rural context and to the needs of rural health professionals and health services. Face-to-face training was considered beneficial because it allowed educators to engage with health professionals when there was a need to use technical research computer programs. Opportunities for face-to-face interaction were often limited due to the amount of travel required in rural areas. Virtual training afforded health professionals with some attendance flexibility although resulted in poor attendance, hampered by poor internet and limited access to information communication technology. A combination of face-to-face and virtual training was suggested:

You put on a lot of face-to-face [training] and they don’t turn up. They [health professionals] say they want online stuff. You put on an online thing and they say they don't want that—they want face-to-face. I think the … millennials have a preference for online. Online works better in terms of time commitments and schedules. I think a mix [of training modes], but with a focus on online, then maybe optional face-to-face, refreshers or stuff like that [could suit]. (Participant 1, MM3)

There's the geographic constraints and the requirement to have to travel or use technology, which we all know can't really be relied upon. (Participant 9, MM2)

Some participants suggested that adaptions to research training required a *complexity* in approach that is yet to be seen in the Victorian context. Future training needed to address the needs of a range of different stakeholders, span a range of durations, cover a broad scope of topics, and use a “just in time” [i.e., opportunity to apply learnings into practice shortly after training] (Participant 13, MM2) approach. This would enable rural health professionals to optimise their participation in research training and apply new knowledge and skills to a relevant issue when needed. Participants suggested future research training could build on locally developed initiatives that have addressed common barriers to training at a health service level. One example of a locally developed and delivered program was a two-day mentored research training program targeted at rurally based health practitioners to support the development of clinically focused research ideas into proposals:

It [training program] was really well structured. Really good presenters and [participants] had to have an idea to begin with. They had to do X amount by the first [workshop], then they had a couple of months between the next [workshop]. There were deadlines, and people had committed [to the tasks]. …People went away, and they had to have a protocol written. Each group had a mentor. Their protocols got critiqued by peers, others that were doing the same program. Then they learned about ethics and that sort of stuff. Now it's up to them to continue it. I know some projects have definitely come from that workshop. (Participant 11, MM2)

Another program provided rural health professionals with mentor-based research education and training through jointly funded research capacity building/translation positions:

[The] positions are … half funded by the health service and half funded by the university. That works better I think because there's an investment on both sides, and [the embedded researchers] are based in the health service. (Participant 2, MM3)

[It’s about] supporting clinicians to do the really simple … projects. …It's a matter of engaging people in reflecting on their practice, reviewing what's been done by them and their team... That's what's going to drive better health care. That's why you want research in health services. (Participant 2, MM3)

Other programs delivered in the region were considered “standalone workshops” and did not require participants to have a specific research idea in mind, or achieve a particular outcome:

The workshops I did were specifically snapshots. So, they were single day, standalone. People who came to the second and the third workshop – we recommended that if you’d been to an earlier workshop that would be good. The first workshop was videoed the first year, so you could watch the video to catch up, that sort of stuff. But mostly they were standalone. (Participant 19, MM5)

Participants acknowledged that preliminary work was required to demystify research activities before engaging rural health professionals in research training. A broad approach supporting significant numbers of health professionals to develop an adequate foundation in research rather than preparing individual health professionals, was suggested. Harnessing an experiential approach by supporting health professionals to participate in research activities and enabling practical experience in posing and answering research questions and observing research impact, was considered important: “if they’ve done a research project or got a poster or a publication or whatever out of it, and they’ve been through the process, then that really encourages them to keep going” (Participant 20, MM3). Other ideas to demystify research were providing regular, regionally focused training pitched at a variety of skill levels, and offering training co-led by health professionals:

In a perfect world, there would be regular education sessions that would be four-hour, workshop-style sessions that would be run weekly. They'd be pitched at different people that are at different levels in terms of their experience, skills and capacity, so we'd have a beginner’s and intermediate [sessions] and [involve] more of your translational researchers or those that are looking at applying for grants. These would just be regular enough that somebody that woke up one morning and decided they wanted to seriously look at doing a research project, they could book in the next week, within the month, and then get started. That would be my dream. We would have clinicians and researchers contributing to those workshops. They would be interactive. There would be built-in milestones. They'd really allow that practical utilisation of the knowledge and skills they pick up. (Participant 9, MM2)

### Outer setting

The policies, structures and processes external to rural health services, such as state government health departments, university policies and health service networks, influenced the development of research training for rural health professionals. Participants acknowledged how the *cosmopolitanism* or collaborative nature and networking between rural health professionals from different regions offered capacity for rural health services to coordinate research training development: “I find working in regional and rural [settings], people are so open to sharing what [training] we have because I guess they know that that's how we get things done” (Participant 14, MM3). However, it was also noted that a lack of strategic coordination may contribute to the ad hoc nature of training, duplication of training and associated costs, and inhibit development of a critical mass of rural health professionals engaged in research activities:

I think the co-ordination structures of [the] Victoria health [department] make it difficult because there is no structured co-ordination between the health services. …If [one health service] decided they really wanted to upskill some researchers and they wanted to put on training, they might only get five people attend so it's not worth their while. To co-ordinate that with several other health services takes a lot of effort. The advantage of Queensland having area health service type model means that you can pull people from several different hospitals. (Participant 1, MM3)

Participants found some *external policies and incentives* advantageous because they allowed research training to occur in worktime: “time to get away to attend the half-day session or a day session is not such a huge issue with the EBA [enterprise bargaining agreement] entitlements and what we offer with some of our programs” (Participant 3, MM2). Others, however, believed some external practices, particularly university processes, impinged upon health professionals’ engagement in training to build research capacity: “when you’re learning to be a nurse, a physio and OT, it’s not your primary aim; your primary aim is to look after hands-on patients. So, therefore these other academic heavy processes that we throw at them are not really of much interest” (Participant 6, MM4). Participants also acknowledged that universities and professional peak bodies could provide further incentives to support rural health services to provide research training opportunities. A suggestion was to ensure close ties between research training and any formal research degrees being undertaken by health professionals or through provision of professional development points:

They [general practitioners] get the CPD points for attending different training and events and that sort of thing. How about maybe thinking about receiving points for publishing manuscripts or that sort of thing? There's a lot of talk about trying to get GPs to be more involved in research and it hasn't been successful yet with any sort of strategy. (Participant 16, MM4)

### Inner setting

Barriers relating to attending metropolitan-based training, meant rural health services largely determined the nature and implementation of research training available to rural health professionals. The *priority* *for training was relative* to other staff demands, including clinical practice and other educational commitments. As a result, participants explained that the delivery of research training intensives often fell short of supporting health professionals to develop useful research skills: “They [health services] invest in individuals, put them through this week-long course. At the end of it [they think] ‘they'll be good to go.’” (Participant 2, MM3). Poor *leadership engagement* resulted in reduced management support for training and poor understanding of the value associated with staff developing and using research skills:

[I] offered that kind of [training] numerous times, face-to-face, over video conference, anything like that. But the hospitals won't allow their staff the time out to do it. That's all it is, that they have to give them the time out to do it. I'm not asking them to pay me for it or anything. (Participant 5, MM5)

Participants suggested some of the barriers experienced at the health service level could be overcome by increasing awareness of *information about research training*, via a training calendar accessible to all rural health services. Another was developing a research training model with an emphasis on dissemination, *organisational incentives* and recognition of training outcomes:

I'd probably go to a bunch of different health services and offer to support one team through one small project. Each of my colleagues, we could take one project each and support them through it. That way other teams in that same health service would see what comes out of it—that they’ve done this thing, it's improved this care. They've gone to a conference, and they've got all this recognition from the CEO or the board. (Participant 2, MM3)

### Characteristics of individuals

Successful implementation of training requires interest and uptake by rural health professionals. Participants described universal individual characteristics that can lead to hesitance amongst rural health professionals and impact the uptake of implemented rural research training. Some participants described a complex relationship between individual characteristics and training participation. For instance, health professionals drew on their *knowledge and beliefs* about the benefits of completing training, and their overall *stage of change* toward being interested and engaged in research. The significant time barriers associated with completing research as a health professional, to decide whether to undertake training was also reported: “it’s a many-pronged beast. … of course, the straight answer is yes, if it’s [training] available, there’s an opportunity. How much investment that’s worth when you’ve still got all the issues around research attitudes, lack of time…” (Participant 6, MM4). These characteristics, in particular time pressures, highlighted the need for training to be available in manageable, cohesive training segments rather than the delivery of ad hoc training topics.

### Process

Participants emphasised the importance of strategically *planning* research training. The planning needed to involve development of context-specific training content. Some participants suggested exploring existing training before developing new programs: “There might already be all the resources we need available, it’s just a matter of identifying them and linking them to the people who need those resources” (Participant 13, MM2). Participants described the short-term *evaluative processes* used to gauge training participants’ perceptions of training and acknowledged the difficulty determining the longer-term training outcomes: “[From] the feedback that I got from the day of [training], some attendees just thought that it was valuable. What the ongoing outcomes of that [training] were, I'm not 100 per cent certain” (Participant 8, MM4). Participants described several processes to improve *engagement* in research training, including selecting appropriate research trainers as champions and providing health professionals with support beyond initial training sessions to apply new research skills:

The skills are great to teach, but they need to be taught in the context of perhaps being involved in a project so they can apply those skills straight away... If you learn about research methodology and then it's six months before you get to apply it, then it's like starting all over again, isn't it? (Participant 16, MM4).

Embedding research trainers within health services was considered a useful approach to further engage health professionals in research training. Participants explained that these roles required experienced researchers with broad skillsets to mentor health professionals undertaking a variety of studies:

That tailored support [is helpful], both in terms of [research] skill but also some [research trainers] function as collegial support as well, because it’s hard work and it’s a rollercoaster, participating in research. (Participant 3, MM2)

The use of research networks, both internal and external to health services, to provide longer-term research support, was described as an important strategy for building research capacity and capability:

They'd have mentors. There would be communities of practice that would be established from each of those so that once people start to progress, they've got people that they can lean out on, because invariably different challenges come up—particularly with frontline clinicians doing research—that you can't cover in a workshop. People need support to get through. Yeah, that would be my ideal. (Participant 9, MM2)

Finally, participants emphasised the need for rural health professionals and researchers to work as a team, to collaborate, to co-drive research training development and delivery: “There's absolute value in having chairs and academics included in the team, but if the ultimate goal is to better engage and empower clinicians to be active researchers, then they need to see it happen with and by clinicians” (Participant 15, MM3).

Table 2 provides a list of characteristics identified by participants to consider for the design and implementation of a coordinated rural research education and training model for rural health professionals in Victoria.

**Table 2:** Rural health research training implementation characteristics

Design components
-Address the varying educational needs of health professionals -Select appropriately trained research educators to mentor and engage health professionals throughout the research process -Provide health professionals with support beyond initial training sessions to apply new research skills -Embed research educators with broad research and education skills sets within health services -Consider jointly funded research translation positions between universities and health services -Record and share workshop content -Tap into existing rural research/practice networks, and establish and maintain communities of practice -Co-design and co-deliver training with rural health professionals -Incentivise research education through university and peak body recognition of training participation e.g., formal qualifications, CPD points
Education and training mechanisms
-Adopt a combination of face-to-face and virtual training – regular, regionally focused training sessions for different skills levels -Provide training in a range of durations -Use a “just in time” approach – specific training is delivered as the health professionals need it -Adopt longer term education and training programs -Set realistic deadlines for health professionals to conduct research projects -Use peer review and mentors -Increase awareness of research training via a training calendar accessible to all rural health services
Training content
-Cover a broad scope of topics, e.g., identifying a research question and applying for ethics approval -Encourage health professionals to identify research projects that address issues relevant to their organisations and/or communities

## Discussion

This study provides unique insights into the design and implementation characteristics of research education and training for rural health professionals from highly informed participants, including rural health managers, rural health academics/research coordinators, and university sector directors/managers. The analysis revealed that a lack of tailoring and strategic coordination for the rural context and tensions between research opportunities and clinical practice are inhibitors to rural health professional engagement in research education and training. The findings suggest a need for a coordinated approach in rural areas, underpinned with characteristics including experiential learning, flexible modes of delivery and use of research networks that provide training opportunities in a timely manner for rural health professionals. These are key areas that need addressing in the design and implementation of future research training for rural health professionals.

The ad hoc, inconsistent, inaccessible, and externally sourced research training courses offered to rural health professionals stifled both their ability to take up training opportunities and to apply to research in the rural context. Research training was often irrelevant and mismatched to the needs of rural health professionals and required rural health professionals to travel significant distances and away from their practicing contexts, which discouraged rural health professionals’ participation. This may result in research being seen as a “dirty word” [15] and contribute to their hesitance to engage in research. Contextually developed training may help to ensure it is delivered in a way that is acceptable to rural people and communities. Consistent with other aspects of rural health, the lack of understanding and integration of the Australian rural context is an inhibitor to driving equitable access across the wide spectrum of services (including research training) for rural communities [37]. Findings of this study suggest that ad hoc training opportunities are unlikely to develop sufficient research competence among rural health researchers to enable confident research engagement and may hamper future efforts to build positive research cultures amongst rural health professionals. Future rural health research training models need to consider well-planned and coordinated, rural context-specific research training.

There is much work to do to develop a consistent approach to rural health research training in the state of Victoria, and in some other Australian jurisdictions. A coordinated rural health research training model could draw from characteristics identified in this study (see Table 2) and in international research. Findings from this study suggest a future model should be widely accessible, based on explicit effort to demystify research and prepare rural health professionals with a foundational understanding of research, and include strategically planned training content that draws from new and existing resources. The model could be designed to: be flexible to the educational needs of health professionals, varying widely in terms of discipline, experience, and research experience and skills; be incorporated within a broader research education program that enables health professionals to apply new skills in practice, and; involve embedded research educators to mentor health professionals throughout the research process [12, 38]. The model could tap into existing research and practice networks and maintain communities of practice to facilitate contextually specific research in a timely manner [39], and incentivise engagement in research education by encouraging universities, peak bodies, and health services to recognise training participation and dissemination outcomes [9]. Within the broader educational model, research training should be co-designed and co-delivered with rural health professionals and adopt a blended model of delivery offered across a range of durations and frequencies ensuring timely availability when required by health professionals [24]. Information about training could be shared via an online training calendar accessible to all rural health professionals. Training content could cover a broad scope of topics, encourage health professionals to identify research topics and engage them in a range of research activities. The development and delivery of training should be informed by adult learning theory and encompass multiple pedagogical approaches to maximise outcomes and impact [10].

The research education and training characteristics identified in this study are somewhat reflective of international and other Australian jurisdictional rural research education and training characteristics. The RRCBP in rural NSW is a theoretically informed, experiential training program that combines mentoring and didactic teaching on core research topics [4]. The AH-TRIP in Queensland is an online statewide training and mentoring program founded on adult learning theory to support health professionals engaging in research translation [20]. In Canada, the *6for6* rural research capacity building program for rural physicians was informed by a set of guiding principles and theories and was co-designed with rural physicians and physician scholars [40, 41]. The *6for6* program facilitates skill development through small group experiential learning and problem-solving [41]. These programs provide working examples of a strategic, coordinated approach to building research capacity in rural areas that incorporates training with characteristics that have been identified in the current study. Our findings and the literature highlight the importance of training that facilitates experiential learning and supports health professional engagement with research that is close to practice.

The lack of systematic planning and coordination of rural health professional research training in Victoria broadly reflects the rural health literature indicating minimal investment in rural health research relative to community need [42], with only 2.4% of all NHMRC funding going towards rural health research between 2005 and 2014, despite approximately 30% of the population living in rural Australia [6]. The metro-centric nature of health research has been identified by policy makers as one of the potential contributors to the health disparities between metropolitan and rural Australia [43]. Our study findings demonstrate, through the perspectives of rural health stakeholders, that similar factors are likely to be impacting on the design and implementation of research training in rural areas, including a lack of investment, and the application of metro-centric models to underpin training. Just as there are gaps in the scientific evidence related to the rural context, research education and training for rural communities follows a similar trend, further inhibiting the generation of contextually specific rural research and potentially advantaging metropolitan areas who receive more context specific training.

Significant financial investment into rural health research education and training models is required to ensure a widely accessible and coordinated research education and training model is designed and implemented drawing on key characteristics identified in this study: rurally designed and delivered for rural health professionals; founded on the innate collaborative ways of rural working, with training content that has sufficient flexibility to be able to be place based; organisationally targeted, locally-relevant, experiential and multidisciplinary in nature. With the development of a government led, state-wide strategic health research plan occurring in Victoria, integrating these principles into the formal plan would be an important first step towards implementing equitable and coordinated rural research training opportunities. University Departments of Rural Health (UDRHs), funded through the federal RHMT program, have supported the implementation of rural research education and training for some time [8]. Further funding could enable UDRHs to undertake the development of a rurally tailored research education and training model. The functioning of UDRHs varies in response to their own regional and rural contexts, however developing an overarching model could accommodate for locally relevant research education and training needs. Academic health science centres, which have provided substantial support in metropolitan areas and seen huge growth in health professional research capacity [44], could contribute to this work in rural areas given their role in fostering collaborations between health services, higher education, and supporting health research and education to promote research translation [45].

### Strengths and limitations of the study

This study included the perspectives of 20 key participants embedded in rural health services across the Australian state of Victoria, resulting in a highly informed sample for this context. The use of an existing theoretical framework ensured that context specific concepts were thoroughly interrogated within each of the participant responses. The findings are applicable to the Victorian context, although learnings may inform future research and developments in other states of Australia. Another limitation was the lack of health professional researchers from the private sector, policymakers, representatives from advocacy and funding bodies (such as the National Rural Health Alliance), and health professionals and other stakeholders from Modified Monash Model category 6 in Victoria that might have provided further insights into rural research training design and implementation characteristics. Further research is needed to explore perspectives of stakeholders outside of the health services system that could influence research training in rural areas, with further sampling needed across all states and territories of Australia.

## Conclusions

This study described the current design and implementation characteristics of research training for rural health professionals in Victoria, Australia that could be used to inform the development of a widely accessible coordinated rural research and education training model. The current approach to research education and training is ad hoc in nature and largely inaccessible for rural health professionals, limiting opportunities for rural health professionals to conduct rural health research and inform practice. This study found the nature of the source and cost of research training prevented momentum in research capacity and capability development. Policies, structures, and processes external to rural health services both enabled training opportunities (e.g., when coordinated by rural health service networks) and stifled opportunities when strategic coordination of training was absent. Health services and individual health professionals experienced a constant tension in priority for research training and other work demands. Research training activities lacked longer-term evaluation. Successful rural health research training that increases quality and quantity of relevant rural health research requires an approach that celebrates and harnesses rural contexts; an approach that is strategic, coordinated, flexible and timely, and reflective the needs of rural health professionals, rural health services and rural communities.

## Data Availability

The datasets generated and analysed during the current study are not publicly available due not having consent from participants to publish but are available from the corresponding author on reasonable request.

## Abbreviations

RHMT: Rural Health Multidisciplinary Training program
WAHTN: Western Australian Health Translation Network
NHMRC: National Health and Medical Research Council
AH-TRIP: Allied Health – Translating Research into Practice
RRCBP: Rural Research Capacity Building Program
AHCEN: Allied Health Clinical Educators Network
AHRT: Allied Health Research and Translation
CFIR: Consolidated Framework for Implementation Research
MM: Modified Monash category
NHS: National Health Services
UDRHs: University Departments of Rural Health
